# Time‐Controlled Refrigerated Stem Cell Therapy Mitigates Scleroderma Fibrosis via Modulation of Mitochondrial Autophagy and Gut Metabolism

**DOI:** 10.1002/advs.202515505

**Published:** 2026-03-31

**Authors:** Xue Xia, Chenfei Kong, Xiaoming Zhao, Naixu Shi, Jinlan Jiang, Ping Li

**Affiliations:** ^1^ Department of Rheumatology and Immunology China‐Japan Union Hospital, Jilin University Changchun China; ^2^ Scientific Research Center China‐Japan Union Hospital, Jilin University Changchun China; ^3^ Department of Stomatology China‐Japan Union Hospital of Jilin University Changchun China

**Keywords:** gut microbiota, intestinal content metabolomics, mesenchymal stem cells, mitophagy, targeting specificity

## Abstract

Scleroderma is a chronic autoimmune disease characterized by progressive fibrosis, associated with high morbidity and mortality, limited therapeutic efficacy, and significant systemic side effects. Therefore, there is an urgent need to develop novel treatment strategies with improved tissue targeting and safety profiles. In this study, a refrigerated‐treated mesenchymal stem cell (RT‐MSCs) system was established by culturing human umbilical cord‐derived MSCs under controlled low‐temperature conditions. The antifibrotic effects of RT‐MSCs were evaluated through both in vivo and in vitro experiments, together with an assessment of their regulatory role in mitochondrial autophagy. Their lesion‐targeting capacity was also investigated. Furthermore, the effects of RT‐MSCs on gut microbiota composition and metabolic pathways in model mice were comprehensively analyzed using 16S rRNA sequencing and intestinal content metabolomics, and the safety of RT‐MSCs was systematically evaluated. The results demonstrated that RT‐MSCs effectively attenuated fibrosis progression by modulating mitochondrial autophagy. Within 30 min after administration, RT‐MSCs accumulated at lesion sites and persisted for up to 7 days. RT‐MSCs significantly improved intestinal microbiota dysbiosis in scleroderma mice and regulated the expression of associated intestinal metabolites. In summary, as an optimized stem cell‐based therapeutic strategy, RT‐MSCs offer new insights and potential avenues for treating scleroderma.

## Graphical abstract

This study established an RT‐MSCs‐based therapeutic approach for scleroderma in mice. RT‐MSCs attenuated fibrosis by regulating mitochondrial autophagy and restored gut microbiota homeostasis. Metabolomic analyses confirmed recovery of key metabolites, and RT‐MSCs demonstrated favorable lesion targeting and safety profiles.

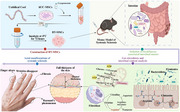

## 1 Introduction

Systemic sclerosis (SSc) is a multisystem autoimmune disease characterized by vascular dysfunction, immune dysregulation, and extensive fibrosis [1]. It is associated with high mortality, with a reported 10‐year survival rate of only 66% [2]. The quality of life of patients with SSc is severely impaired, primarily due to fibrosis‐driven destruction of normal tissue architecture in the skin, lungs, kidneys, and skeletal system, resulting in serious complications such as pulmonary hypertension, acute kidney injury, and acro‐osteolysis [3]. Among these manifestations, scleroderma represents the most prevalent clinical feature of SSc. Current standard treatment for scleroderma relies on combination therapy with methotrexate and corticosteroids; however, these regimens are accompanied by substantial adverse effects [4, 5]. Although novel therapeutic approaches, including biologics, have been introduced, they have failed to halt or reverse disease progression [6]. Therefore, there is an urgent need to develop safe and efficacious therapeutic strategies for scleroderma.

Mesenchymal stem cells (MSCs) derived from the umbilical cord (UC) have been widely used to repair damaged tissues and organs owing to their self‐renewal capacity and multipotent differentiation potential [7]. These cells show broad plasticity, robust proliferative capacity, and convenient accessibility. UC‐MSCs are immunoprivileged and display extremely low tumorigenicity, making them attractive candidates for regenerative medicine and disease therapy. Compared with MSCs from other sources, UC‐MSCs can be obtained in higher yields [8]. Our previous studies further demonstrated that cryopreserved MSCs can serve as efficient drug‐delivery vehicles and exert therapeutic effects in cancer models [9].

Mitochondria are highly dynamic organelles involved in cellular metabolism, survival, necrotic cell death, and programmed apoptosis. Mitophagy, a cytoprotective process, selectively eliminates damaged or superfluous mitochondria and maintains mitochondrial quality control, preserving intracellular homeostasis [10]. Emerging evidence has revealed mechanistic links between mitophagy and various diseases, as well as the therapeutic potential of mitochondrial regulators in animal models [11, 12]. However, research on mitophagy is still in its early stages. While its roles have been explored in cancer [13], cardiovascular [14], and neurodegenerative diseases [15], investigations in autoimmune diseases are still limited.

Accumulating evidence indicates that gut microbiota and their metabolites play critical roles in the pathogenesis of autoimmune diseases (ADs) [16]. Gut dysbiosis has been consistently associated with multiple ADs, including systemic lupus erythematosus (SLE) and rheumatoid arthritis (RA) [17, 18]. Gut microbiota can influence disease progression by modulating host immune responses and inflammatory pathways [19]. For example, butyrate, a microbiota‐derived metabolite, has been shown to alleviate arthritis by regulating both Breg and Treg cell activity [20]. Moreover, microbiota‐host interactions may shape disease outcomes through metabolic pathways, particularly those related to vitamin metabolism and arachidonic acid cascades.

Given the close association between gut microbiota and scleroderma, microbiota modulation has emerged as a promising therapeutic target for SSc. MSCs and their exosomes have demonstrated therapeutic potential in inflammatory diseases by regulating gut microbiota composition and related metabolites [21]. For instance, MSC‐derived exosomes significantly ameliorate inflammatory responses in murine colitis models by remodeling both the structure and function of the gut microbiota [22]. Natural compounds such as ginsenoside Rh2 exert immunomodulatory and anti‐inflammatory effects by regulating gut microbiota via exosome‐mediated mechanisms [23]. These results provide a strong rationale for developing novel microbiota‐targeted therapeutic approaches.

However, whether MSCs can ameliorate scleroderma by modulating gut microbiota and their metabolites remains largely unexplored. Therefore, this study investigates the therapeutic effects of refrigerated‐treated MSCs (RT‐MSCs) in a murine model of scleroderma, with particular emphasis on their interactions with gut microbiota and associated metabolites. Using 16S rRNA sequencing and untargeted metabolomics, we aim to elucidate the mechanisms underlying the microbiota–host interactions underlying RT‐MSCs therapy, providing new theoretical insights and experimental evidence to support their potential application in scleroderma treatment.

## 2 Results

### 2.1 Preparation and Characterization of RT‐MSCs

**FIGURE 1:**
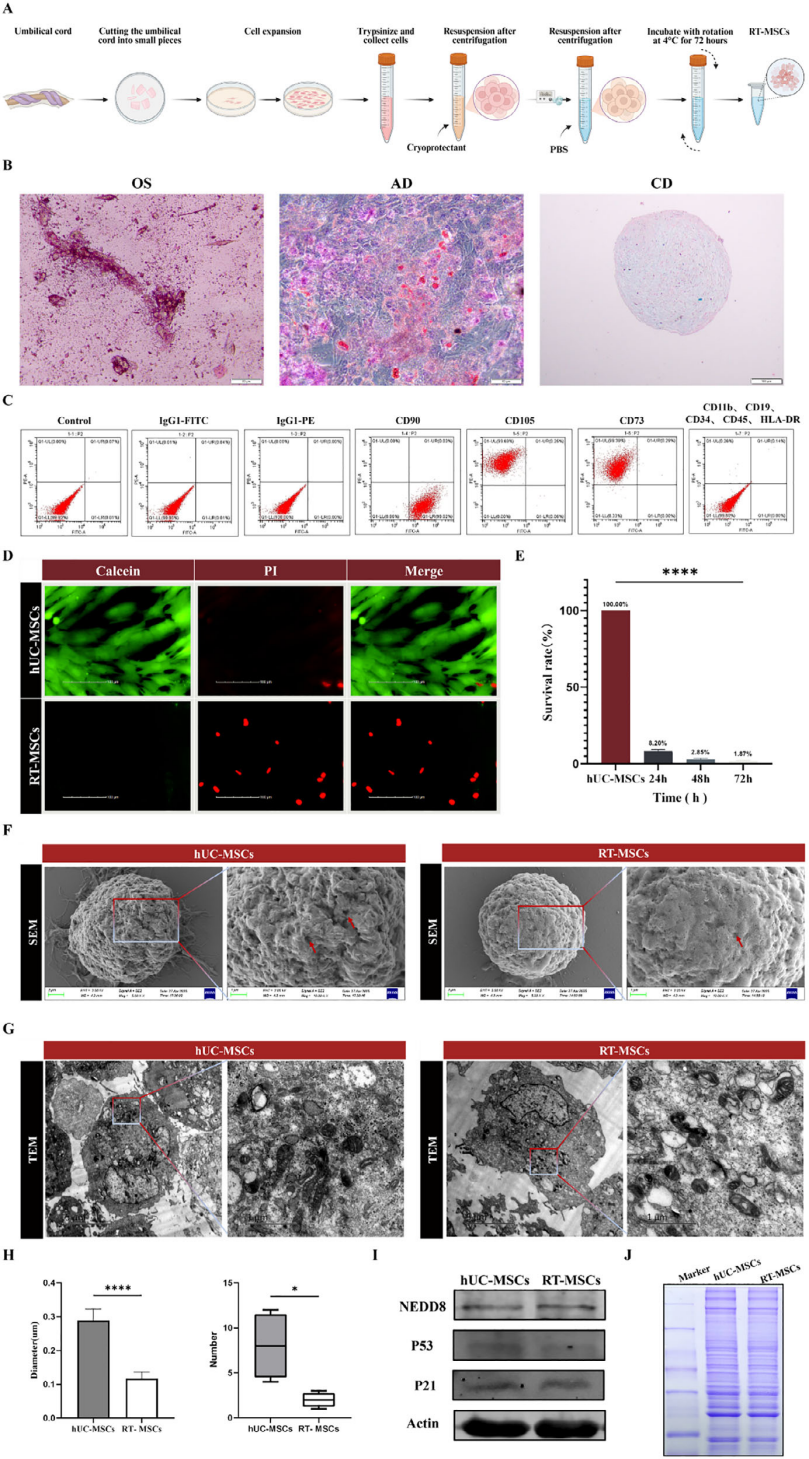
Preparation and Characterization of RT‐MSCs. (A) Schematic diagram of the RT‐MSCs preparation process. (B) Adipo‐osteo‐chondrogenic differentiation. (C) Flow cytometry analysis of the surface markers of MSCs. (D) Double staining with Calcein‐AM/PI of hUC‐MSCs and RT‐MSCs (confocal microscopy; scale bar = 100 µm). (E) CCK‐8 assays of MSCs at different time points under 4°C conditions. (F) SEM image of hUC‐MSCs and RT‐MSCs (scale bars = 2 µm and 1 µm, respectively). Red arrows indicate the diameter of surface pores. (G) TEM image of hUC‐MSCs and RT‐MSCs (scale bars = 5 µm and 1 µm). (H) Data are mean ± s.e.m. *n* = 6. Statistics: two‐tailed unpaired Student's *t*‐test, ^*^ *p* < 0.05, ^****^ *p* < 0.0001. (I) Western blot analysis of NEDD8, p53, and p21 expression in hUC‐MSCs and RT‐MSCs. (J) Coomassie brilliant blue staining.

RT‐MSCs were generated using a controlled low‐temperature protocol in which cryoprotectant and PBS buffer were sequentially added to human umbilical cord‐derived MSCs (hUC‐MSCs), followed by incubation at 4°C for 72 h to obtain RT‐MSCs (Figure 1A). Trilineage differentiation assays confirmed the multipotency of MSCs: osteogenic differentiation resulted in red calcium nodule formation, adipogenic differentiation led to intracellular lipid droplet accumulation, and chondrogenic differentiation produced cartilage spheroids that stained blue with Alcian Blue (Figure 1B). Flow cytometric analysis showed that MSCs were positive for CD90, CD105, and CD73, while negative for CD11b, CD19, CD34, CD45, and HLA‐DR (Figure 1C). Double staining with Calcein‐AM/PI and CCK‐8 assays indicated complete loss of viability in RT‐MSCs (Figure 1D,E). Compared with hUC‐MSCs, scanning electron microscopy revealed that RT‐MSCs retained a spherical morphology without significant changes in cell size but demonstrated smoother surfaces (Figure 1F), accompanied by reduced pore number and diameter (Figure 1H). Transmission electron microscopy (TEM) further confirmed preservation of intracellular organelle structures in RT‐MSCs (Figure 1G). Western blot analysis demonstrated significantly increased NEDD8 expression in RT‐MSCs, accompanied by reduced levels of the cell cycle‐related proteins p53 and p21 (Figure 1I). Coomassie Brilliant Blue staining showed that total protein levels remained stable in RT‐MSCs (Figure 1J).

### 2.2 Validation of RT‐MSCs Targeting Ability

**FIGURE 2:**
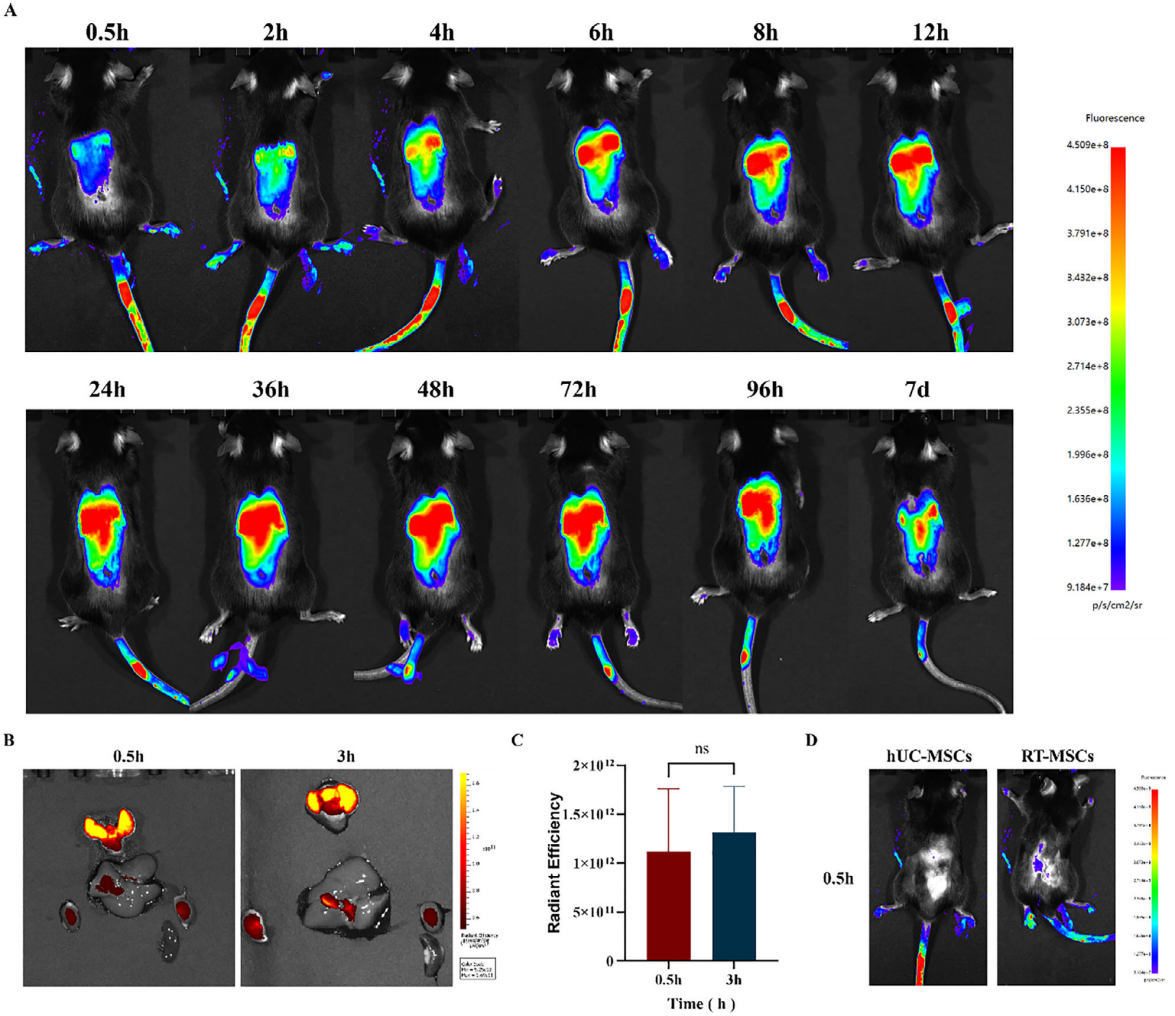
RT‐MSCs exhibit lesion‐targeting capacity in a mouse model of scleroderma. (A) In vitro fluorescence imaging of DiR‐labeled RT‐MSCs. (B) Representative in vivo fluorescence images of a scleroderma mouse after CY5.5‐labeled RT‐MSCs injection. (C) Quantitative analysis of lung fluorescence intensity. Data are mean ± s.e.m. *n* = 3. Statistics: two‐tailed unpaired Student's *t*‐test, ns: no significance. (D) Comparison of the lesion‐targeting capacity between DiR‐labeled RT‐MSCs and DiR‐labeled hUC‐MSCs.

The intrinsic homing capacity of MSCs enables their targeted recruitment to inflammatory sites [24]. However, enhancing their targeting efficiency remains a major challenge in stem cell‐based therapies. To assess targeting specificity, RT‐MSCs were labeled with DiR and administered to scleroderma mouse models via tail vein injection. As shown in Figure 2A, fluorescent signals accumulated at skin lesion sites within 30 min after injection, with signal intensity increasing progressively over time. Consistent results were obtained using CY5.5‐labeled RT‐MSCs, which demonstrated pronounced pulmonary accumulation (Figure 2B,C), with lung fluorescence intensifying in a time‐dependent manner following intravenous administration. Compared with DiR‐labeled hUC‐MSCs, RT‐MSCs displayed significantly enhanced lesion‐targeting capacity (Figure 2D), characterized by more rapid localization to pathological regions. Free DiR dye was injected intravenously into scleroderma mice, and no fluorescence accumulation was observed in skin lesions; signals were detected only at the tail injection site (Figure S1). These results confirm that the fluorescence signals observed at lesion sites originated from labeled RT‐MSCs.

### 2.3 Antifibrotic and Autophagy‐Modulating Effects of RT‐MSCs In Vitro

**FIGURE 3:**
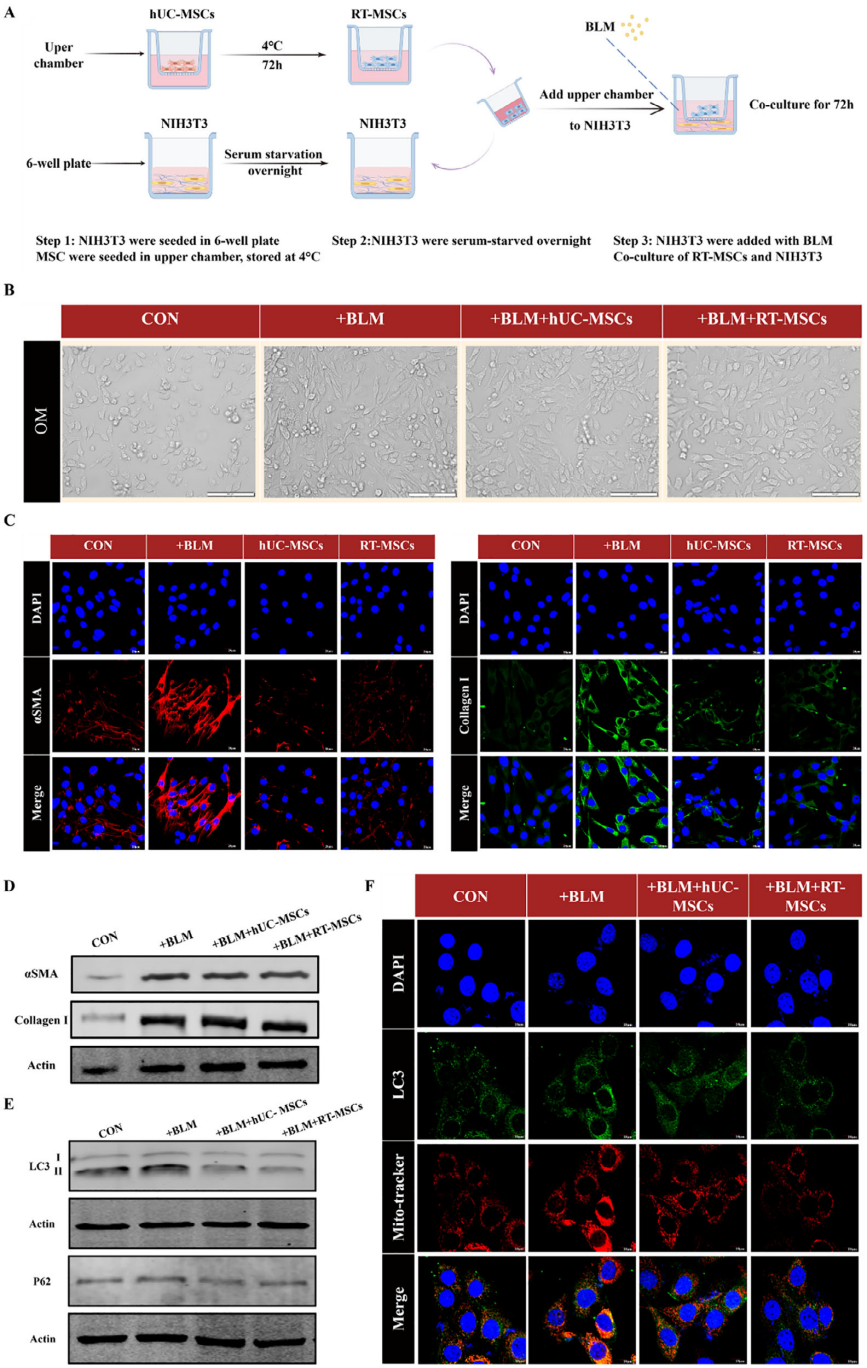
Antifibrotic and autophagy‐modulating effects of RT‐MSCs in vitro. (A) Schematic of the fibroblast activation model. (B) Morphological changes in fibroblasts (scale bar = 100 µm). (C) Immunofluorescence staining of αSMA and collagen I in fibroblasts (scale bar = 20 µm). (D) Western blot analysis of αSMA and collagen I expression. (E) Western blot analysis of autophagy markers (LC3B‐II/I and P62). (F) Immunofluorescence of LC3B and mitochondrial fluorescence probes. (Red: Mitotracker labelled; Green: LC3B; scale bar = 20 µm).

The anti‐fibrotic effects of RT‐MSCs were evaluated using in vitro assays. As illustrated in Figure 3A, fibroblasts were co‐cultured with RT‐MSCs in a Transwell system for 72 h, after which cellular morphology and protein expression were assessed. Light microscopy revealed that untreated fibroblasts demonstrated typical spindle‐shaped morphology with dendritic extensions, whereas BLM‐treated cells appeared flattened and dispersed, with enlarged nuclei. Treatment with RT‐MSCs largely restored normal cellular morphology (Figure 3B). Immunofluorescence analysis demonstrated a significant increase in fibrotic markers following BLM exposure, confirming the successful establishment of a pro‐fibrotic model. In comparison, fibroblasts co‐cultured with RT‐MSCs showed significantly reduced expression of fibrosis‐associated proteins, including αSMA and collagen I (Figure 3C), indicating a strong anti‐fibrotic effect. These results were further validated by Western blot analysis (Figure 3D). For comparison, mitochondria and cell membranes isolated from hUC‐MSCs were also evaluated. They showed weaker antifibrotic activity than those from RT‐MSCs (Figure S2), further supporting the efficacy of the RT‐MSCs preparation.

Autophagy‐related pathways were investigated to elucidate the underlying mechanisms. BLM treatment significantly increased autophagic activity in fibroblasts, as reflected by higher levels of autophagy‐associated markers. Following RT‐MSC intervention, the LC3II/I ratio was significantly reduced (Figure 3E), indicating effective attenuation of excessive autophagy. Moreover, immunofluorescence analysis revealed colocalization of autophagy‐related proteins with mitochondrial fluorescent probes (Figure 3F), suggesting that mitophagy plays a critical role in the RT‐MSCs‐mediated alleviation of fibrosis.

### 2.4 Antifibrotic and Autophagy‐Modulating Effects of RT‐MSCs In Vivo

**FIGURE 4:**
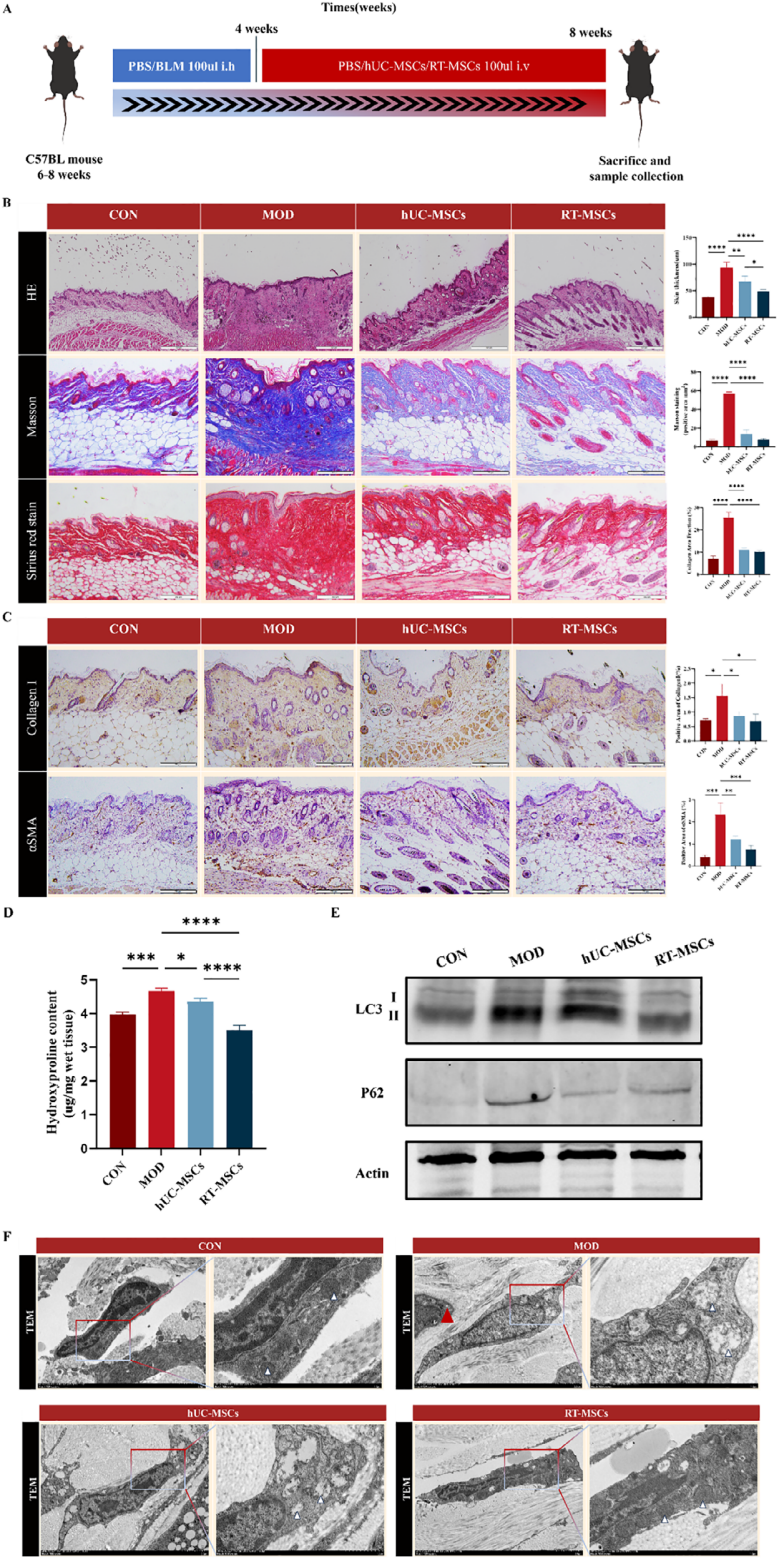
RT‐MSCs alleviate skin fibrosis and modulate mitophagy in a mouse model of scleroderma. (A) Schematic of experimental timeline. (B) H&E, Masson's trichrome, and Sirius red staining (scale bar = 200 µm). (C) Collagen I and αSMA immunohistochemistry (scale bar = 200 µm). (D) Hydroxyproline Assay. (E) Immunoblot analysis of the LC3B‐II/I ratio and P62 levels. (F) Transmission electron micrographs of skin (Red triangles indicate autophagolysosomes, scale bar = 2 µm). Data are mean ± s.e.m. *n* = 6. Statistics: Multiple groups were assessed via one‐way ANOVA with Tukey's post hoc test for multiple comparisons. ^*^ *p* < 0.05, ^**^ *p* < 0.01, ^***^ *p* < 0.001 ^****^ *p* < 0.0001.

The therapeutic efficacy of RT‐MSCs was assessed in a murine model of skin fibrosis. A scleroderma model was established by subcutaneous BLM injection, followed by intravenous administration of the indicated treatments via the tail vein (Figure 4A). Skin tissues were harvested at week 8 for histopathological evaluation. Hematoxylin and eosin (H&E) staining revealed significant skin thickening in the model group, while Masson's trichrome staining demonstrated substantial collagen accumulation, confirming successful establishment of the scleroderma model. RT‐MSCs treatment significantly attenuated these pathological changes (Figure 4B). Immunohistochemical analysis further showed reduced expression of the fibrotic markers αSMA and collagen I following RT‐MSCs administration (Figure 4C). Hydroxyproline assays indicated a significant increase in collagen content in the skin of model mice, which was significantly decreased after RT‐MSCs treatment, further confirming the robust antifibrotic capacity of RT‐MSCs (Figure 4D). Further, immunohistochemical staining of lung tissues revealed high αSMA expression in the model group, whereas RT‐MSCs treatment significantly reduced this signal (Figure S3). These results suggest that subcutaneous BLM administration induces not only skin fibrosis but also pulmonary fibrotic changes, which RT‐MSCs can partially alleviate.

Transmission electron microscopy of skin tissues demonstrated vacuolated mitochondria in fibroblasts from model mice. Although hUC‐MSCs treatment alleviated fibrosis, it did not restore mitochondrial morphology. In comparison, RT‐MSCs administration significantly improved mitochondrial ultrastructure, returning it to a near‐normal state (Figure 4E). Western blot analysis of skin tissues further showed that RT‐MSC treatment significantly downregulated autophagy‐related markers, consistent with the in vitro findings and TEM observations (Figure 4F). These results indicate that RT‐MSCs may exert their antifibrotic effects by modulating mitophagy and influencing fibroblast activation.

### 2.5 RT‐MSCs Regulate Mitophagy

**FIGURE 5:**
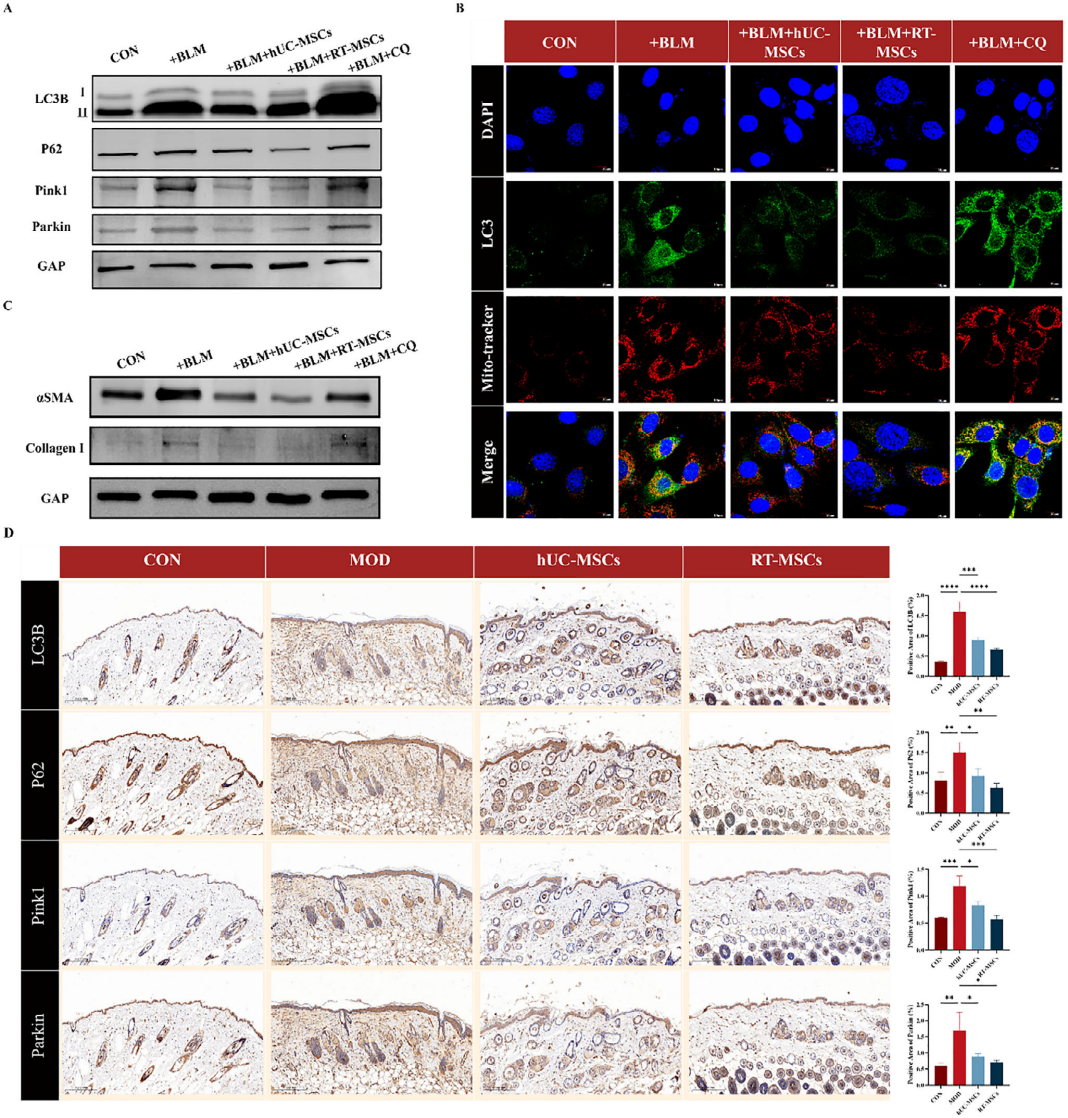
RT‐MSCs Regulate Mitophagy. (A) Western blot analysis of mitophagy‐related proteins in fibroblasts treated with chloroquine (CQ). (B) Immunofluorescence quantification of the LC3B (II/I) ratio in fibrotic cells with or without CQ treatment. (C) Western blot analysis of fibrosis‐related proteins in fibroblasts treated with CQ. (D) Immunohistochemical staining and quantification of LC3B, P62, Pink1, and Parkin in skin tissues from different groups. Data are mean ± s.e.m. *n* = 6. Statistics: Multiple groups were assessed via one‐way ANOVA with Tukey's post hoc test for multiple comparisons. ^*^ *p* < 0.05, ^**^ *p* < 0.01, ^***^ *p* < 0.001 ^****^ *p* < 0.0001.

To further clarify the role of mitophagy in fibrosis, chloroquine (CQ) was employed to inhibit mitophagic flux. In the fibrotic cell model, CQ treatment increased the LC3B II/I ratio and promoted P62 expression, accompanied by upregulation of the mitophagy‐associated proteins Pink1 and Parkin (Figure 5A). These results were further supported by immunofluorescence analysis, which demonstrated a significant increase in the LC3B II/I ratio following mitophagy inhibition (Figure 5B). Blockade of autophagy led to a significant reduction in fibrosis‐related protein expression (Figure 5C), suggesting that inhibition of mitophagy attenuates fibrotic progression. Immunohistochemical staining revealed significantly promoted Pink1 and Parkin expression in scleroderma model mice, whereas RT‐MSCs treatment substantially reduced the levels of both proteins (Figure 5D). These results indicate enhanced mitophagy in the disease model and suggest that RT‐MSCs may exert their anti‐fibrotic effects by modulating mitophagy.

### 2.6 Regulatory Effects of RT‐MSC on Gut Microbiota and Metabolites in a Mouse Model of Scleroderma

#### 2.6.1 BLM‐Induced Fibrosis Model Alters Gut Microbiota Composition

**FIGURE 6:**
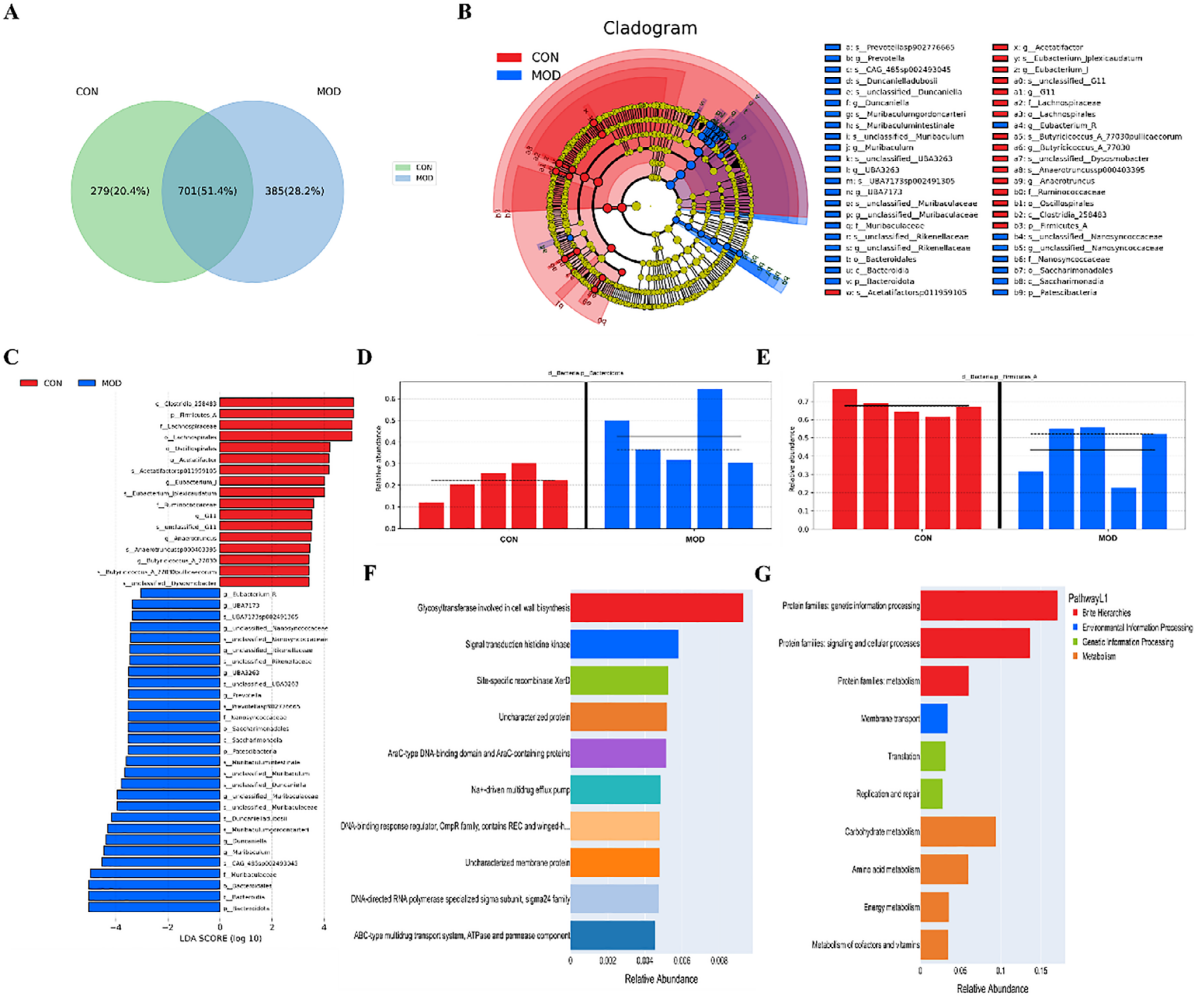
Gut microbiota alterations in BLM‐induced fibrotic mice. (A) Venn diagram of ASVs between the control and model groups. (B) Taxonomic clustering of the microbial communities. (C) LEfSe analysis identifying differentially enriched taxa (LDA score >2). (D, E) Relative abundance of Bacteroidota and Firmicutes at the phylum level. (F) COG functional classification of the MOD group. (G) KEGG pathway enrichment analysis of the MOD group. *n* = 5.

Gut dysbiosis disrupts immune function and metabolic homeostasis, contributing to the development of multiple diseases. To examine the involvement of gut microbiota in scleroderma, the intestinal microbial composition in BLM‐induced scleroderma mice was profiled. 16S rRNA sequencing of fecal samples identified 279 and 385 unique amplicon sequence variants (ASVs) in the control and MOD groups, respectively, with 701 ASVs shared between the two groups (Figure 6A). Differentially abundant taxa were determined using linear discriminant analysis effect size (LEfSe). At the phylum level, MOD mice showed increased abundance of Bacteroidota and reduced abundance of Firmicutes. At the genus level, Muribaculum and Duncaniella were significantly enriched in the MOD group, whereas Clostridia, Lachnospiraceae, and Lachnospirales predominated in control mice (Figure 6B,C). These compositional differences were further validated by subsequent analyses (Figure 6D,E). Functional prediction revealed improved expression of glycosyltransferases involved in cell wall biosynthesis and histidine kinases in fecal samples from the MOD group (Figure 6F). Moreover, KEGG pathway enrichment analysis indicated significant alterations in genetic information processing, carbohydrate metabolism, and amino acid biosynthesis pathways (Figure 6G).

#### 2.6.2 Alterations in Gut Microbiota Composition and Structure Mediated by RT‐MSCs

**FIGURE 7:**
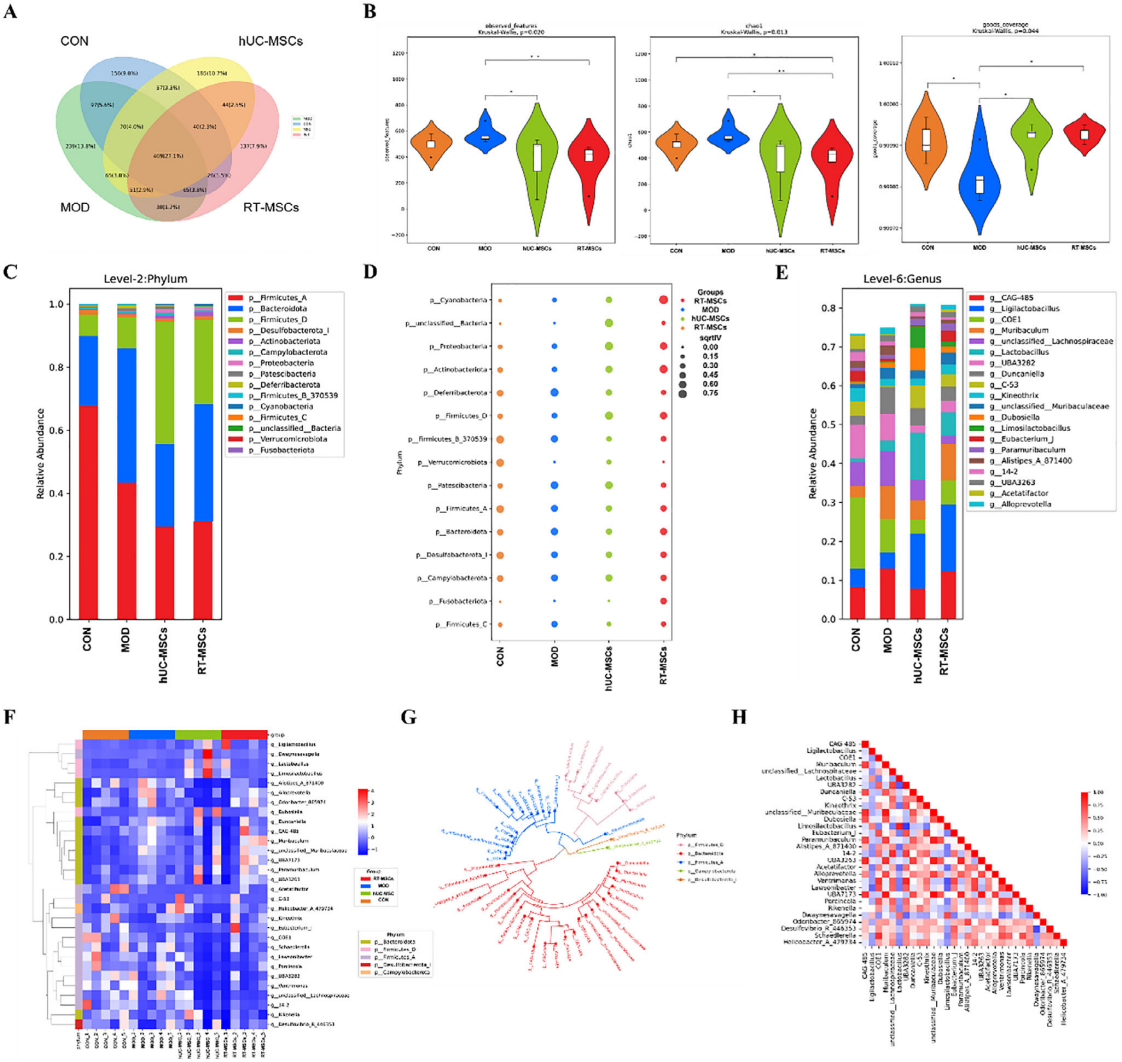
RT‐MSCs‐mediated restructuring of gut microbiota composition and architecture. (A) Venn diagram of the ASV distribution. (B) α‐Diversity indices of the gut microbiota. (C) Phylum‐level taxonomic composition. (D) Differential abundance of microbial taxa across the groups. (E) Genus‐level taxonomic profiling. (F) Hierarchical clustering heatmap of genus‐level abundances (red: high; blue: low). (G) Phylogenetic tree of the gut microbial communities. (H) Spearman correlation heatmap of interspecies relationships. Data are mean ± s.e.m. *n* = 5. Statistics: Multiple groups were assessed via one‐way ANOVA with Tukey's post hoc test for multiple comparisons. ^*^ *p* < 0.05, ^**^ *p* < 0.01.

To evaluate the regulatory effects of RT‐MSCs on gut microbiota in bleomycin‐induced scleroderma mice, fecal samples from each experimental group were collected for 16S rRNA sequencing. The number of unique ASVs identified in the control, model, hUC‐MSCs, and RT‐MSCs groups was 156, 239, 185, and 137, respectively, with 469 ASVs shared among all four groups (Figure 7A), indicating pronounced alterations in microbial composition across treatments. Alpha diversity analyses were conducted to assess microbial richness and diversity. Compared with the MOD group, the RT‐MSCs group showed reduced Observed species and Chao1 indices, reflecting decreased species richness, whereas increased Goods coverage suggested restoration of overall microbial community functionality (Figure 7B). Phylum‐level profiling identified five dominant taxa, Firmicutes, Bacteroidota, Desulfobacterota, Actinobacteriota, and Patescibacteria (Figure 7C). Indicator species analysis of the top 30 taxa revealed significant increases in Proteobacteria and Actinobacteriota following RT‐MSCs treatment (Figure 7D), accompanied by decreases in Patescibacteria and Deferribacterota. Further genus‐level analysis showed that RT‐MSCs intervention reversed the high abundances of Lachnospiraceae and Duncaniella observed in the model group (Figure 7E,F). Phylogenetic tree analysis indicated that these genera primarily belonged to Firmicutes and Bacteroidota (Figure 7G). These results suggest that RT‐MSCs restore the relative abundances of Firmicutes, Bacteroidetes, Proteobacteria, and Actinobacteria toward levels observed in healthy controls. Genus‐level correlation analysis identified strong associations between *Paramuribaculum* and *Muribaculum*; *Dysosmobacter* and *Ventrimonas*; *Lawsonibacter* and *Enterococcus*; and *Acutalibacter* and *Schaedlerella* (Figure 7H), suggesting potential synergistic interactions among Firmicutes, Proteobacteria, and Actinobacteriota.

#### 2.6.3 BLM‐Induced Fibrotic Model Triggers Gut Metabolic Reprogramming

**FIGURE 8:**
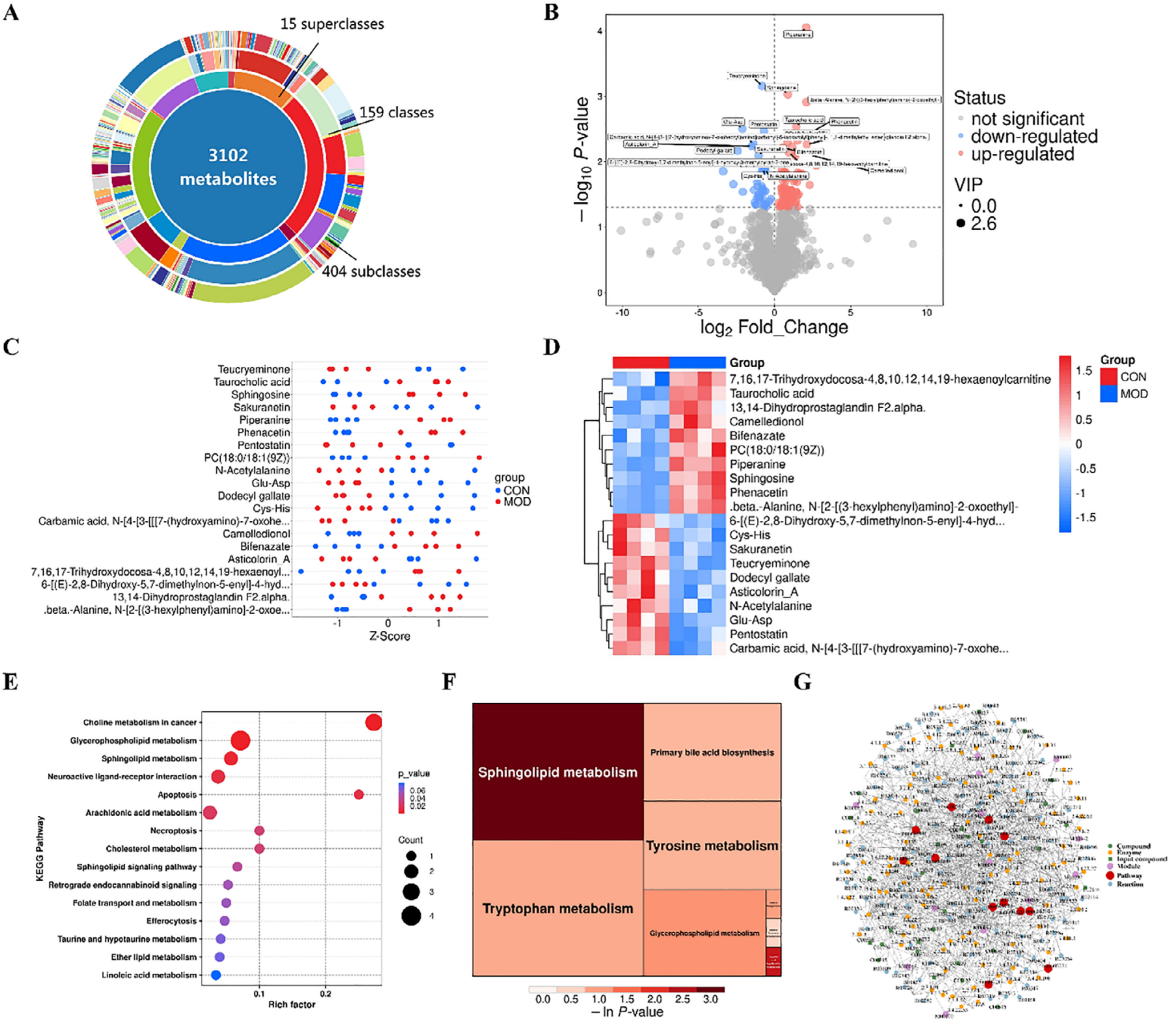
BLM‐induced metabolic reprogramming of the intestinal microenvironment. (A) Circular taxonomy plot of metabolite classification. (B) Volcano plot of the differential metabolites. (C) Z‐score heatmap of the differential metabolites. (D) Hierarchical clustering analysis of metabolite profiles. (E) KEGG pathway enrichment classification. (F) Treemap visualization of the metabolic pathways. (G) Regulatory network analysis of metabolic interactions. *n* = 4.

As shown in Figure 8A, a total of 3102 metabolites were identified and categorized into 15 superclasses, 159 main classes, and 404 subclasses, highlighting the complexity of the gut metabolome and indicating the broad metabolic impact of BLM exposure and RT‐MSC intervention. Differential metabolite analysis revealed 74 metabolites significantly upregulated and 43 downregulated considerably in the BLM‐treated group compared with controls (Figure 8B). Following Z‐score normalization across samples, the top 10 upregulated and downregulated metabolites were selected for statistical evaluation (Figure 8C). Higher metabolites included 7,16,17‐trihydroxydocosa‐4,8,10,12,14,19‐hexaenoylcarnitine, taurocholic acid, and 13,14‐dihydroprostaglandin F2α, which are implicated in essential fatty acid metabolism and pathological compensatory pathways, fat digestion and cholesterol metabolism, as well as signal transduction, oxidative stress, and inflammatory responses, respectively. In comparison, the significant reduction of Cys‐His suggests potential BLM‐induced suppression of immunoregulatory and antioxidant mechanisms (Figure 8D,E).

To further elucidate the biological relevance of these metabolic perturbations, KEGG pathway enrichment analysis was performed on the differentially expressed metabolites. Significant enrichment was observed in pathways related to choline metabolism in cancer, glycerophospholipid metabolism, sphingolipid metabolism, neuroactive ligand–receptor interaction, apoptosis, necroptosis, and arachidonic acid metabolism. These pathways involve coordinated interactions among lipids, amino acid derivatives, and proteins, and are closely linked to cell membrane remodeling and apoptotic regulation. Such metabolic reprogramming may underlie BLM‐mediated systemic regulation of skin fibrosis by modulating cellular signaling, inflammatory responses, and intestinal epithelial barrier integrity. In particular, sphingolipid metabolism, tryptophan metabolism, primary bile acid biosynthesis, and glycerophospholipid metabolism showed especially strong associations with the identified differential metabolites (Figure 8F). Based on these findings, an integrated network enrichment model incorporating metabolic pathways, functional modules, enzymatic reactions, and metabolite profiles was constructed, systematically depicting the interactive metabolic landscape under BLM challenge (Figure 8G). Network analysis revealed convergence at several key nodes, suggesting that BLM promotes fibrotic progression by coordinating the regulation of this interconnected metabolic network.

#### 2.6.4 Microbiome‐Metabolite Interactions

**FIGURE 9:**
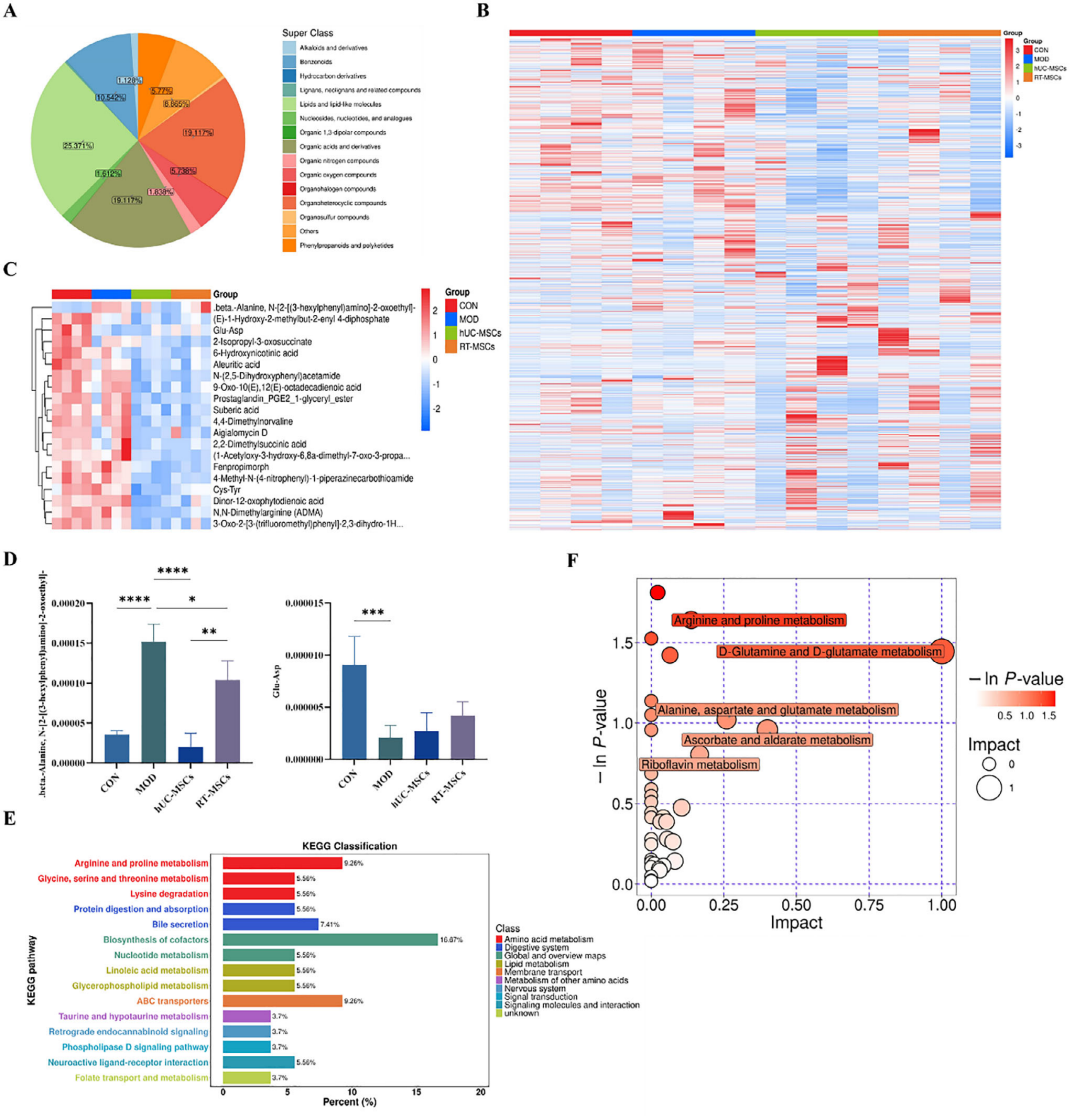
Microbiome‐metabolite profiling (A) Pie chart of metabolite categories across the CON, MOD, hUC‐MSCs, and RT‐MSCs groups. (B) Hierarchical clustering heatmap of fecal metabolite profiles (*n* = 4). (C) Zoomed‐in view of the top 20 differentially abundant metabolites (relative levels). (D) Differential expression of β‐alanine and Glu‐Asp across groups. Data are mean ± s.e.m. *n* = 4. Statistics: Multiple groups were assessed via one‐way ANOVA with Tukey's post hoc test for multiple comparisons. ^*^ *p* < 0.05, ^**^ *p* < 0.01, ^***^ *p* < 0.001 ^****^ *p* < 0.0001. (E) KEGG pathway classification of differential metabolites. (F) Metabolic pathway impact analysis.

Metabolomic profiling of mouse fecal samples revealed a diverse array of metabolites, with lipids and lipid‐like molecules (25.371%), organic acids and derivatives (19.117%), and organoheterocyclic compounds (19.117%) representing the predominant classes (Figure 9A). Heatmap analysis demonstrated pronounced differences in metabolite distributions among experimental groups (Figure 9B). The top 20 most differentially abundant metabolites were subsequently selected for focused analysis (Figure 9C), revealing increased β‐alanine levels and decreased glutamate–aspartate dipeptide (Glu–Asp) in the MOD group compared with controls; importantly, this trend was reversed following RT‐MSC treatment (Figure 9D). KEGG enrichment analysis of the differential metabolites revealed significant enrichment in pathways related to cofactor biosynthesis, membrane transport, and arginine/proline metabolism (Figure 9E). Integrated pathway analysis further identified arginine/proline metabolism and D‐glutamine/D‐glutamate metabolism as the most highly enriched pathways, demonstrating strong correlations with the observed metabolic alterations (Figure 9F).

#### 2.6.5 RT‐MSCs‐Mediated Regulation of Gut Metabolites in the Scleroderma Mouse Model

**FIGURE 10:**
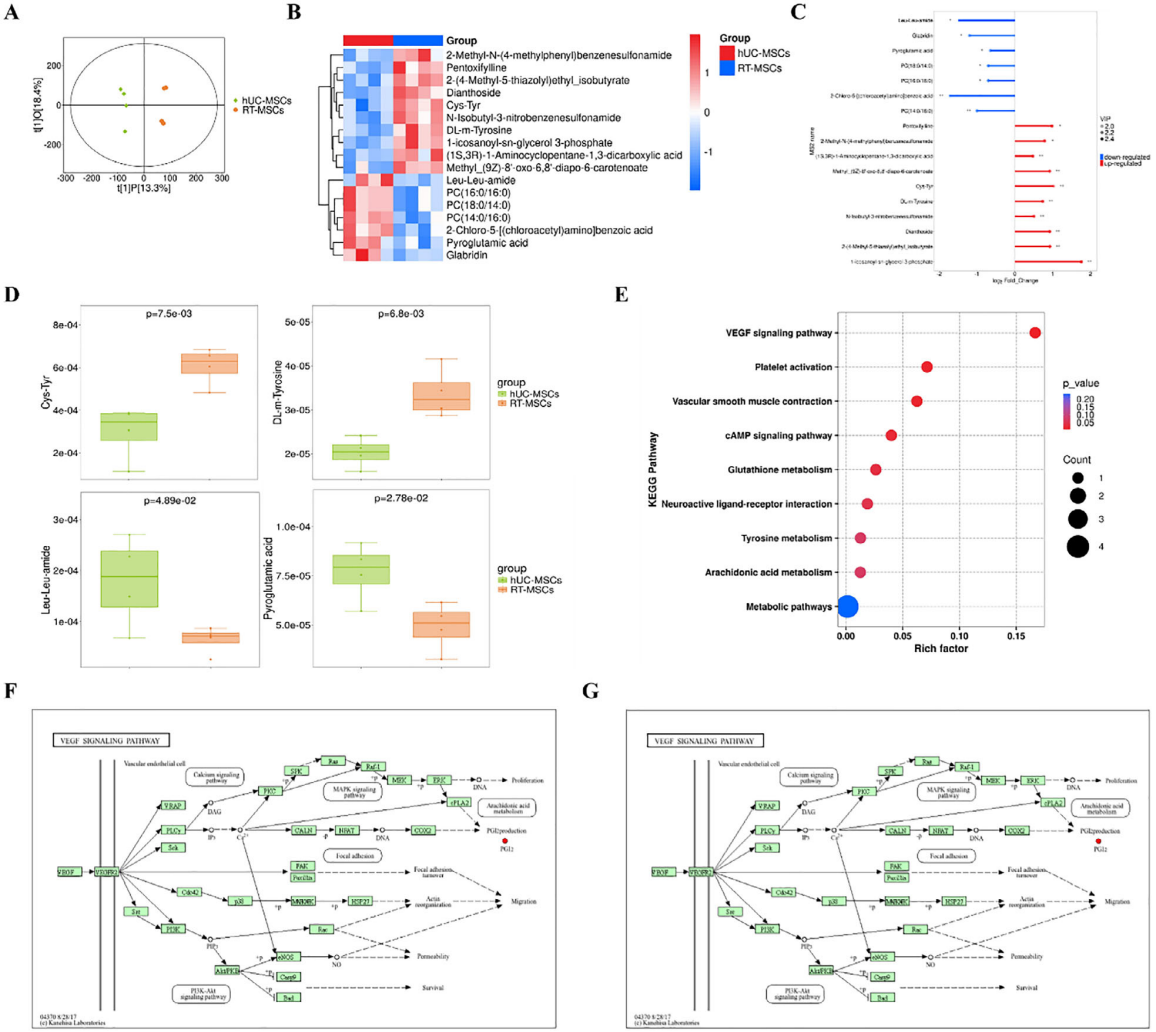
Metabolomic profiling of intestinal contents in hUC‐MSCs and RT‐MSCs treated groups. (A) PCA of fecal metabolomes. (B) Heatmap visualization of metabolite profiles. (C) Volcano plot of the differential metabolites. Data are mean ± s.e.m. *n* = 4, Statistics: two‐tailed unpaired Student's *t*‐test. (D) Comparative analysis of individual amino acid concentrations. (E) KEGG pathway enrichment analysis. (F, G) KEGG pathway enrichment analysis of the significantly enriched metabolic pathways.

OPLS‐DA analysis of fecal metabolites revealed clear separation between the hUC‐MSCs and RT‐MSCs treatment groups, despite both being stem cell‐based therapies, indicating distinct metabolic modulation by RT‐MSCs (Figure 10A). Multivariate statistical analysis identified 1174 differential metabolites, including 976 upregulated and 198 downregulated species. Given the biological relatedness and potential co‐regulation of metabolites within shared pathways, hierarchical clustering was performed on the top 10 most significantly upregulated and downregulated metabolites (Figure 10B,C). Compared with hUC‐MSCs treatment, RT‐MSCs administration increased considerably levels of eicosanoids, purines and purine derivatives, carbohydrates, and carbohydrate conjugates, while reducing glycerophosphocholines. Further pathway‐focused analysis revealed that mark was significantly elevated in cysteinyltyrosine and DL‐m‐tyrosine, accompanied by decreased levels of leucyl‐leucinamide and pyroglutamic acid (Figure 10D). KEGG pathway enrichment analysis demonstrated significant activation of vascular endothelial growth factor signaling, platelet activation, vascular smooth muscle contraction, the cAMP signaling pathway, and glutathione metabolism in the RT‐MSCs group (Figure 10E). KEGG mapping of differential metabolites further showed upregulation of the anti‐inflammatory lipid mediator PGI2 and downregulation of the collagen‐associated amino acid 5‐oxoproline (Figure 10F,G), suggesting that RT‐MSCs may mitigate skin fibrosis by suppressing inflammation and reducing collagen synthesis.

#### 2.6.6 The Role of Arginine and Proline Metabolism in the Fibrosis Process

Based on our gut metabolomics analyses, arginine and proline metabolism emerged as pathways closely associated with fibrotic progression. To further elucidate the contribution of this metabolic axis to fibrosis, both in vivo and in vitro studies were performed.

**FIGURE 11:**
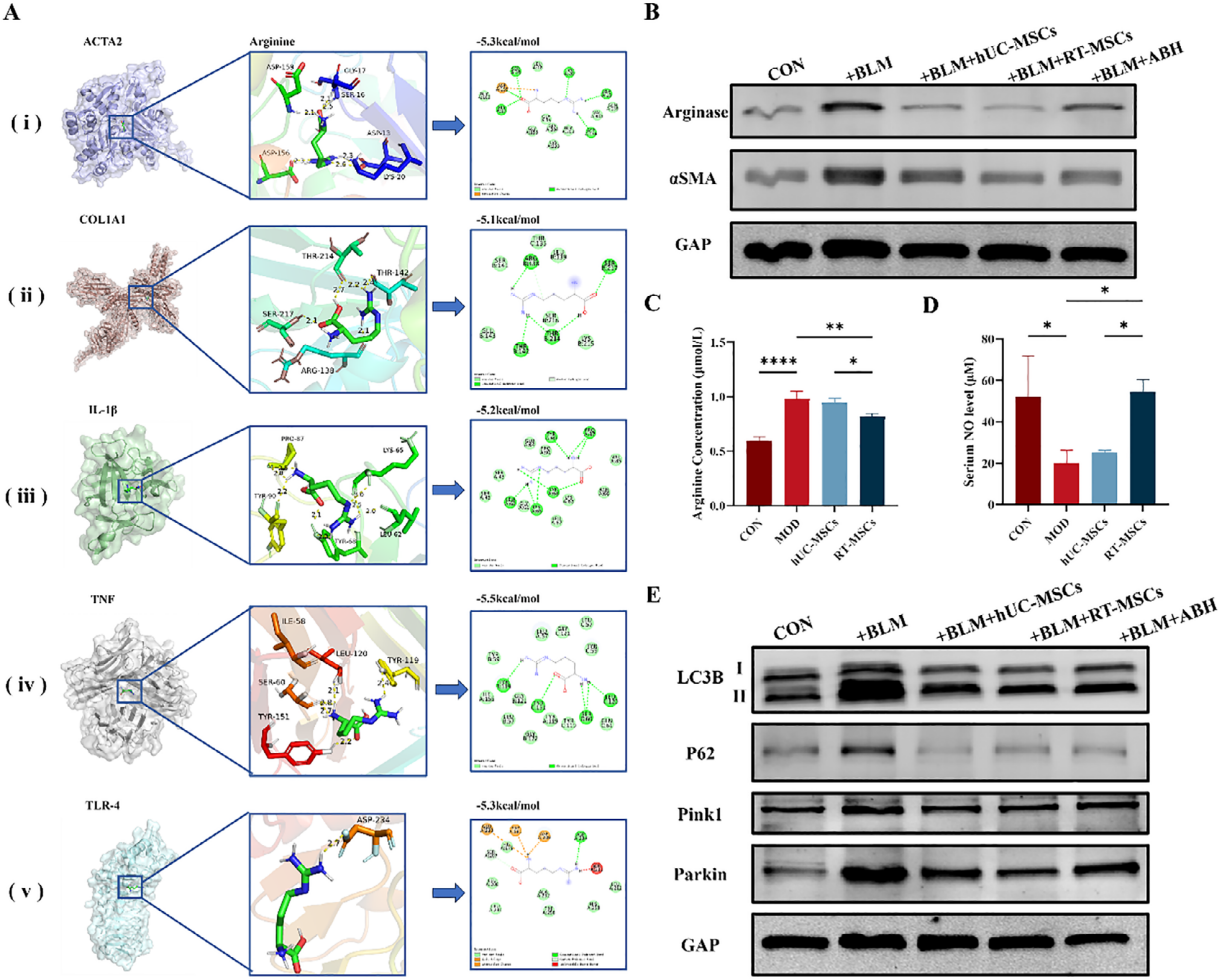
Role of arginine in fibrosis and its regulation by RT‐MSCs. (A) Molecular docking of arginine with fibrosis/inflammation targets (ACTA2, COL1A1, IL‐1β, TNF, and TLR4). The binding energies were below −5 kcal/mol. (B) Western blot analysis showing fibrosis‐related protein and arginase expression after arginine inhibition treatment. (C) Arginine levels in the mouse serum. (D) NO levels in mice after RT‐MSCs treatment. Data are mean ± s.e.m. n = 6. Statistics: Multiple groups were assessed via one‐way ANOVA with Tukey's post hoc test for multiple comparisons. *p < 0.05, **p < 0.01, ***p < 0.001 ****p < 0.0001. (E) Western blot analysis of mitophagy‐related protein expression after arginine inhibition treatment.

First, potential systemic sclerosis–related targets were identified through public database screening (Figure S4), and high‐confidence targets were selected for molecular docking with arginine and proline. Docking results showed that arginine bound with binding energies below−5 kcal/mol to fibrosis‐associated markers (ACTA2, COL1A1) and inflammation‐related proteins (IL‐1β, TNF, TLR4), indicating strong affinity for the active sites of these targets (Figure 11A). Similarly, proline showed binding energies below −4.25 kcal/mol with the same fibrosis‐ and inflammation‐associated proteins, suggesting effective interaction with their active pockets (Figure S5). These results support potential regulatory roles for arginine and proline in fibrotic processes.

To further define the mechanistic involvement of arginine in fibrosis, validation experiments were conducted in both cellular and animal models. Western blot analysis revealed that arginine inhibition significantly reduced the expression of fibrosis‐related proteins and arginase in the fibrotic cell model (Figure 11B). Similarly, quantification of arginine levels in mouse serum showed a significant increase in the scleroderma model group, whereas RT‐MSCs treatment significantly decreased arginine concentrations (Figure 11C). Moreover, nitric oxide (NO) assays indicated that following RT‐MSC intervention, arginine metabolism was redirected toward the NO synthesis pathway in the fibrosis model (Figure 11D). These results demonstrate that the arginine metabolic pathway plays a key role in disease progression and suggest that RT‐MSCs may mitigate fibrosis by modulating arginine metabolic pathways. We also examined the expression levels of mitophagy‐related proteins. The results showed that modulation of the arginine metabolic pathway significantly alleviated the activation of bleomycin‐induced mitophagy (Figure 11E). These findings suggest that arginine metabolism plays a key regulatory role in mitophagy.

### 2.7 Safety Evaluation of RT‐MSCs

**FIGURE 12:**
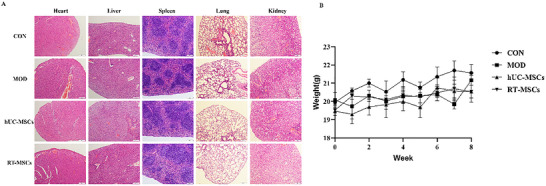
Safety validation of RT‐MSCs. (A) H&E staining of the mouse heart, liver, spleen, lung, and kidney. (B) Changes in the body weights of mice in the different treatment groups (*n* = 6).

Given that clinical translation is the ultimate objective of material development, safety was a primary focus of this study. Following euthanasia, major organs, including the heart, liver, spleen, lungs, and kidneys, were harvested for H&E staining. As shown in Figure 12A, neither hUC‐MSCs nor RT‐MSCs treatment produced detectable toxic effects in these vital organs. No significant changes in body weight were observed throughout the experimental period across all groups (Figure 12B), further supporting the favorable safety profile of RT‐MSCs.

## 3 Discussion

This study demonstrates that RT‐MSCs significantly attenuate fibrosis in a murine model of scleroderma and show promising feasibility, therapeutic efficacy, and safety. Compared with synthetic carriers, this cell‐based delivery platform offers distinct targeting advantages and effectively circumvents biological barriers [25]. hUC‐MSCs inherently possess homing capabilities comparable to those of bone marrow‐derived stem cells and preferentially localize to immune‐associated lesions, rendering them attractive vehicles for the treatment of autoimmune diseases. However, hUC‐MSCs are susceptible to reduced stability during storage, transport, and in vivo retention. Moreover, achieving precise lesion targeting while preserving transient structural integrity remains a major challenge. To overcome these limitations, we propose an innovative approach using RT‐MSCs. These cells have lost proliferative activity yet retain key therapeutic functions, enabling stable, targeted, and effective fibrosis mitigation.

Ubiquitination plays a central role in innate immune regulation by rapidly modulating inflammatory signaling pathways to ensure effective immune responses while preventing excessive activation [26]. NEDD8, a ubiquitin‐like modifier, regulates multiple biological processes, including protein localization, stability, and activity [27]. The C‐terminus of p53 can be modified by NEDD8 [28], and p53 activation induces p21, which suppresses tumor growth by inhibiting cyclin‐dependent kinase complexes, proliferating cell nuclear antigen, and transcription factors [29]. In this study, RT‐MSCs showed significantly raised NEDD8 expression, accompanied by reduced p53 and p21 levels, suggesting that low‐temperature treatment promotes ubiquitination‐related modifications while relieving p21‐mediated cell cycle arrest, influencing RT‐MSC functional properties.

Mitophagy is a critical cytoprotective mechanism that maintains mitochondrial network homeostasis by selectively clearing damaged or redundant mitochondria [10]. It plays essential roles in inflammation, metabolic remodeling, and cell fate determination [12]. Previous studies indicate that patients with systemic lupus erythematosus may demonstrate impaired mitophagy, manifested as defective mitochondrial trafficking to lysosomes or abnormal degradation, leading to the pathological accumulation of mitochondrial DNA [30]. Moreover, genetic variants in autophagy‐related genes are strongly associated with increased susceptibility to autoimmune diseases [31, 32], suggesting that mitophagy dysfunction may represent a shared pathological feature across multiple ADs [33]. In SSc, skin fibrosis develops through chronic inflammation involving fibroblast mitophagy. Our results indicate that RT‐MSCs may alleviate fibrosis by modulating mitophagy in fibroblasts. Transmission electron microscopy revealed a higher mitochondrial content in RT‐MSCs than in hUC‐MSCs, suggesting that RT‐MSCs exert therapeutic effects by transferring mitochondria to fibroblasts; however, this hypothesis requires further experimental confirmation.

Tianyuan Ci et al. reported that liquid nitrogen‐treated acute myeloid leukemia (AML) cells enhanced doxorubicin delivery to bone marrow and functioned as a cancer vaccine, prolonging survival in AML mouse models [34]. Compared with AML cells, RT‐MSCs offer superior safety, lower immunogenicity, and improved lesion‐targeting capability. RT‐MSCs also facilitate storage and transportation while preserving stem cell functionality. If utilised as a delivery platform, RT‐MSCs have the potential to rapidly localise to scleroderma skin lesions, thereby improving drug delivery and reducing systemic toxicity. Furthermore, RT‐MSCs persist at lesion sites for up to 2 weeks, demonstrating sustained, targeted retention, underscoring their promise as an effective delivery vehicle.

The gut microbiome, the largest microbial ecosystem in the human body, plays a pivotal role in health and disease, with dysbiosis closely linked to numerous pathological conditions [35, 36]. Previous studies have shown that BLM‐induced fibrotic mice exhibit characteristic alterations in gut microbiota, a significant reduction in Firmicutes abundance [37, 38], mirroring changes observed in SSc patients, who also display decreased abundance of butyrate‐producing bacteria and increased pro‐inflammatory taxa [39]. As Firmicutes constitute the primary butyrate‐producing phylum in the human colon, their depletion may lead to reduced intestinal butyrate levels [40]. Given butyrate's potent immunomodulatory and anti‐inflammatory effects that extend beyond the gut to systemic pathways, diminished Firmicutes abundance in BLM models may contribute to fibrotic progression through butyrate deficiency [41]. Although evidence linking Proteobacteria, Actinobacteria, Patescibacteria, and Deferribacterota to scleroderma remains limited, emerging data suggest their involvement in fibrotic processes. For example, clinical studies have reported significantly reduced levels of Actinobacteria in patients with silicosis and progressive pulmonary fibrosis [42]. These results suggest that RT‐MSCs may restore gut microbial homeostasis through multi‐target regulation, indirectly exerting anti‐fibrotic effects in scleroderma. Future studies should involve larger‐scale samples to further validate the specific molecular targets involved.

KEGG pathway analysis identified enrichment of arginine metabolism during fibrosis progression. Arginine, a conditionally essential amino acid, remains relatively understudied in scleroderma. Its metabolites, including proline and ornithine, are significantly increased in lung tissues from patients with idiopathic pulmonary fibrosis and in BLM‐induced murine models [43]. Pulmonary fibroblasts depend on arginine to activate TGF‐β‐mediated mTORC1 signaling, sustaining collagen synthesis, and arginine also contributes to the formation of a pro‐fibrotic signaling microenvironment beyond its role in protein synthesis [44]. Arginine methylation regulates mitophagy [45], suggesting that fibrosis may be modulated through arginine‐driven alterations in mitochondrial energetics and autophagic processes. Recent evidence further highlights epigenetic reprogramming as central to idiopathic pulmonary fibrosis (IPF) pathogenesis, with glutamine metabolism regulating anti‐apoptotic gene expression in fibroblasts via histone methylation, emphasizing glutaminolysis in epigenetic control [46]. Our results demonstrate that arginine plays a critical role in fibrosis. Targeted regulation of this pathway not only effectively alleviates the severity of fibrosis but also reduces the level of mitochondrial autophagy, suggesting a potential functional correlation between the two. However, the precise mechanisms by which amino acids such as arginine and glutamine regulate fibrosis remain incompletely defined. Future studies integrating skin transcriptomics and additional multi‐omics approaches may help identify key molecular targets.

In RT‐MSCs–treated groups, pathways related to eicosanoid metabolism, purine metabolism, and purine derivatives were significantly enriched. Accumulating evidence indicates that inhibition of eicosanoid degradation, particularly prostaglandins, attenuates fibrosis by suppressing fibroblast proliferation and myofibroblast differentiation [47]. Meanwhile, dysregulated purine nucleotide metabolism promotes epithelial‐mesenchymal transition by disrupting serine biosynthesis pathways [48]. These findings provide mechanistic insight into the multifaceted anti‐fibrotic actions of RT‐MSCs.

## 4 Conclusions

RT‐MSCs were developed and applied to a murine model of scleroderma. The results demonstrated that RT‐MSCs significantly alleviated BLM‐induced skin fibrosis and exerted multifaceted therapeutic effects, including modulation of mitophagy, regulation of the skin–gut microbiota axis, and induction of metabolic reprogramming, without evident safety concerns in the short term. These results highlight RT‐MSCs as a promising therapeutic strategy for scleroderma and may also provide valuable insights into the treatment of other autoimmune diseases.

## 5 Experimental Section

### 5.1 Preparation of MSC

The umbilical cord tissue was washed with phosphate‐buffered saline (PBS; Sigma, Germany), minced into fragments (∼3 mm), and cultured in Dulbecco's Modified Eagle Medium (DMEM; StemCell, Canada) with 20% fetal bovine serum (FBS; HyClone, USA). Cells were maintained at 37°C in 5% CO_2_, with the medium changed every 3 days. MSCs were passaged upon reaching 80% confluence. Experiments were performed using P3‐P6 cells cultured in α‐MEM (HyClone, USA) supplemented with 10% FBS and 1% penicillin/streptomycin (HyClone, USA).

### 5.2 Identification of MSCs

MSCs were differentiated into adipocytes (adipogenic medium, Sigma, USA; 18–21 days), osteoblasts (osteogenic medium, Sigma; 28 days), and chondrocytes (chondrogenic medium, Sigma; 21 days), with lineage confirmation by Oil Red O (Solarbio, China), Alizarin Red (Solarbio), and Alcian Blue/Nuclear Fast Red (Solarbio) staining, respectively. Surface markers (CD105/CD90/CD73+; CD11b/CD19/CD34/CD45/HLA‐DR‐; BioLegend antibodies, USA) were verified by flow cytometry.

### 5.3 Preparation and Characterization of RT‐MSCs

First, a cryopreservation solution containing 5% DMSO, α‐MEM, and 20% FBS was prepared, filtered, and pre‐cooled at 4°C for later use. The P3 MSCs were then digested, the reaction was neutralized, and the cells were collected by centrifugation. The cells were gently resuspended in cryopreservation solution to a density of 1 × 10^6^ cells/mL. Next, incubate at 4°C for 10–15 min for DMSO osmotic equilibration. Finally, the samples were centrifuged to remove the cryoprotectant, washed with PBS, resuspended in a low‐temperature maintenance medium, and rotated at 4°C for 72 h. Cell morphology was observed using scanning electron microscopy (SEM) and transmission electron microscopy (TEM; Hitachi, Japan). Changes in surface proteins were detected using Coomassie Brilliant Blue staining.

### 5.4 Animal Experimental Design

Eight‐week‐old female C57BL/6 mice (Beijing Vital River Laboratory Animal Technology Co., Ltd.) were housed at the Animal Center of Jilin University Basic Medical College under controlled conditions (25°C, 60% humidity). The study protocol was approved by the Animal Ethics Committee of Jilin University (approval no. SY202312017). After one week of acclimatization, scleroderma models were established by subcutaneous injection of 100 µL bleomycin solution (1 mg/mL in PBS) or PBS alone [49, 50]. Four weeks postmodelling, mice were randomly divided into four groups (6 mice per group): model group (PBS), hUC‐MSCs group (3 × 10^5^ cells), RT‐MSCs group (3 × 10^5^ cells), and control group (untreated, PBS), with all treatments administered via tail vein injection.

### 5.5 16S rRNA Amplicon Sequencing

This study utilized Illumina NovaSeq PE250 sequencing to analyze microbial communities. Genomic DNA was extracted, PCR‐amplified with target primers, and processed into libraries through end repair, A‐tailing, and adapter ligation. After size selection (2% agarose gel), sequencing data were processed using DADA2 to generate ASVs, followed by rarefaction normalization. Subsequent analyses included α‐ and β‐diversity, taxonomic composition, differential species, and functional prediction.

### 5.6 Metabolomics Analysis

For polar metabolite analysis, 25 ± 1 mg of sample was homogenized in 500 µL of precooled solvent (methanol:acetonitrile:water = 2:2:1 with internal standards) using beads (35 Hz, 4 min) and ultrasonication (ice‐water bath, 3 cycles). After −40°C incubation (1 h) and centrifugation (12 000 rpm, 15 min, 4°C), the supernatant was analyzed using UHPLC (Vanquish system; ACQUITY BEH Amide column, 2.1 × 50 mm, 1.7 µm). The data were converted (ProteoWizard) and processed using a custom R package with BiotreeDB (v3.0) for metabolite identification.

### 5.7 Western Blot

Fibroblasts (purchased from the Cell Bank of the Type Culture Collection Committee, Chinese Academy of Sciences) were treated with BLM at a concentration of 20 µM for 72 h to induce fibrosis. After cell collection, RIPA lysis buffer containing protease and phosphatase inhibitors was added to extract the total proteins. Fibrosis marker α‐smooth Muscle Actin (αSMA, 1:1000, MedChemExpress, USA), collagen synthesis marker Type I Collagen (Collagen I, 1:100, Proteintech China), and mitochondrial autophagy markers microtubule‐associated protein 1A/1B‐light chain 3 (LC3, 2 µg/ml, Abcam, USA) and Sequestosome‐1 (P62, 1:5000, Abmart, China) were detected.

### 5.8 Histopathological Analysis

Skin tissues were fixed with 4% paraformaldehyde and embedded in paraffin before being sectioned at a thickness of 4 µm. H&E staining, Masson's trichrome staining, and Sirius red staining were performed according to standard protocols to evaluate skin fibrosis. Immunohistochemistry was performed on paraffin sections to detect αSMA (1:50) and Collagen I (1:500), as described in the manufacturer's instructions.

### 5.9 Immunofluorescence

Cells were cultured on coverslips in 24‐well plates. Prior to fixation, live cells were incubated with MitoTracker Red CMXRos (Yeasen, China) for 30 min at 37°C. Subsequently, cells were fixed with 4% paraformaldehyde for 15 min at room temperature and permeabilized using 0.1% Triton X‐100 for 10 min. Nonspecific binding was blocked with 5% BSA in PBS for 30 min. Cells were then incubated with the LC3 antibody (1:200) at 4°C overnight, followed by a secondary antibody. Finally, nuclei were stained with DAPI (Boster, China) for 5 min at room temperature. Protein expression and localisation were observed and imaged using a confocal fluorescence microscope.

### 5.10 Inhibition of Mitophagy

To elucidate the role of mitophagy in the observed phenotypic effects, we employed a pharmacological approach to inhibit the process. During bleomycin treatment, the cells were concurrently exposed to the mitophagy inhibitor chloroquine (10 µM, MedChemExpress).

### 5.11 Intervention in the Arginine Metabolic Pathway

To investigate the role of the arginine metabolic pathway in fibrosis, we pharmacologically inhibited it in a cell model. The specific procedure was as follows: while inducing fibrosis with bleomycin, the arginine inhibitor ABH hydrochloride (100 µM, MedChemExpress) was added to the culture medium.

### 5.12 NO Assay

Serum (50 µL) was added to the microplate wells, followed by the sequential addition of equal volumes of Griess Reagent I and II to each well (Beyotime, China). After mixing, the plate was incubated at room temperature in the dark for 10–15 min. Finally, the absorbance was measured at 540 nm, and the concentration of NO metabolites in the serum (µM) was calculated based on the standard curve.

### 5.13 Arginine Content Assay

Standard solutions, diluted samples, and deionized water (as blank control) were added to a 96‐well plate (Solarbio). The detection working solution was then added to each well, followed by incubation on ice in the dark for 20 min. Subsequently, the stop solution was added, and the absorbance was immediately measured at 525 nm using a microplate reader. Finally, the arginine concentration (µmol/L) in the samples was calculated based on the standard curve.

### 5.14 Statistical Analysis

Data were analyzed using GraphPad Prism 9.0. Two‐group comparisons were performed using a two‐tailed unpaired Student's *t*‐test. Multiple group comparisons were performed using one‐way ANOVA. Results are expressed as mean ± SEM, with *p *< 0.05 considered statistically significant.

## Author Contributions

X.X., C.K., and P.L. designed the study. X.X. performed the experiments, collected the data, and analyzed and interpreted the data. All authors contributed to the writing of the manuscript, discussed the results and implications, and edited the manuscript at all stages.

## Conflicts of Interest

The authors declare no conflicts of interest.

## Supporting information




**Supporting File**: advs75110‐sup‐0001‐SuppMat.docx.

## Data Availability

The data supporting the findings of this study are available upon request from the corresponding author. The data are not publicly available due to privacy or ethical restrictions.
