# Supplementary material for: Time‐Controlled Refrigerated Stem Cell Therapy Mitigates Scleroderma Fibrosis via Modulation of Mitochondrial Autophagy and Gut Metabolism
Source: Adv Sci (Weinh). 2026 Mar 31;13(34):e15505. doi: 10.1002/advs.202515505 (PMC13285162; doi:10.1002/advs.202515505)
Supplement: Supplementary file 1 — Supporting File: advs75110‐sup‐0001‐SuppMat.docx. [file ADVS-13-e15505-s001.docx]

Supplementary material

**Time-controlled refrigerated stem cell therapy mitigates systemic sclerosis fibrosis *via* modulation of mitochondrial autophagy and gut metabolism**

Xue Xia^1,#^, Chenfei Kong^2,#^, Xiaoming Zhao^2^, Naixu Shi^3^, Jinlan Jiang^2,*^, Ping Li^1,*^

1 Department of Rheumatology and Immunology, China-Japan Union Hospital, Jilin University, Changchun, 130033, China

2 Scientific Research Center, China-Japan Union Hospital, Jilin University, Changchun, 130033, China

3 Department of Stomatology, China-Japan Union Hospital of Jilin University, Changchun, 130033, China

*Corresponding author: Jinlan Jiang, jiangjinlan@jlu.edu.cn; Ping Li, [li_ping@jlu.edu.cn](mailto:li_ping@jlu.edu.cn)

#These authors contributed equally to this work.

**Supplemental Figures**

**
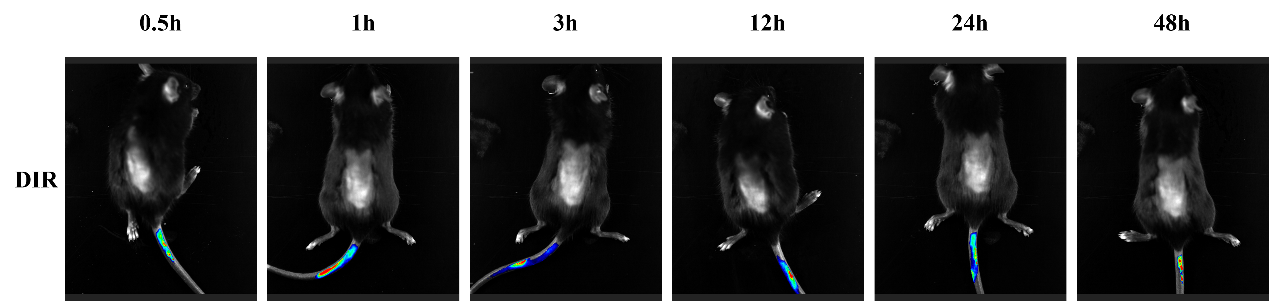
**

Figure. S1. Free DiR dye alone produced detectable signals only at the tail injection site, confirming that the fluorescence signals observed at the lesion sites originated from the labelled RT-MSCs.

**
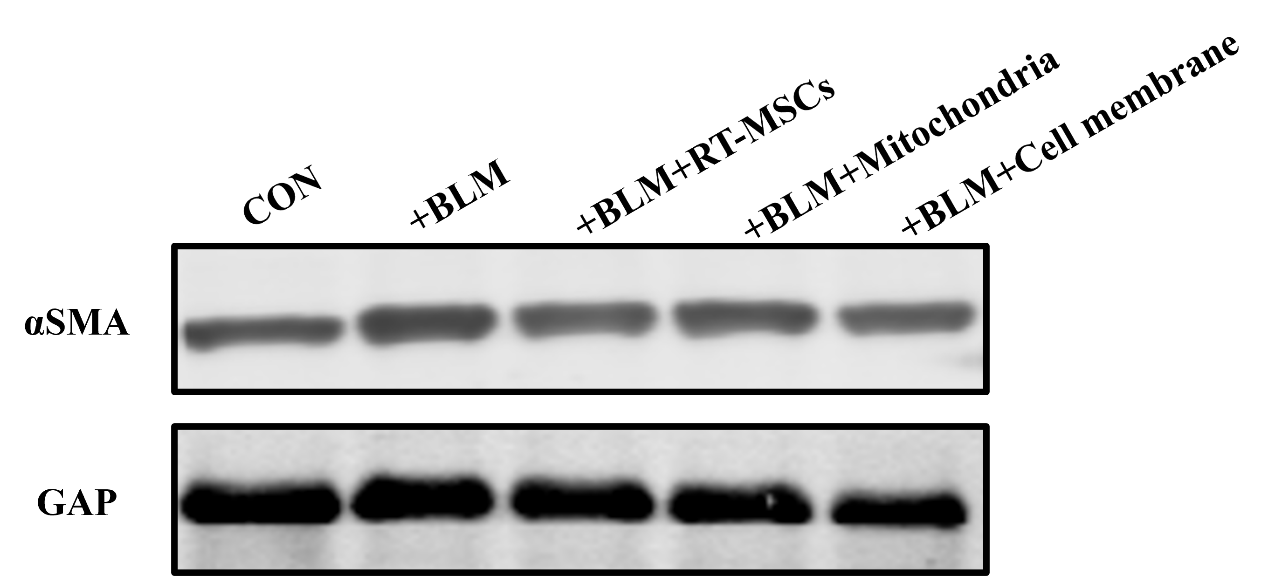
**

Figure. S2. hUC-MSC-derived mitochondria and cell membranes exhibited reduced anti-fibrotic activity compared to those isolated from RT-MSCs.

**
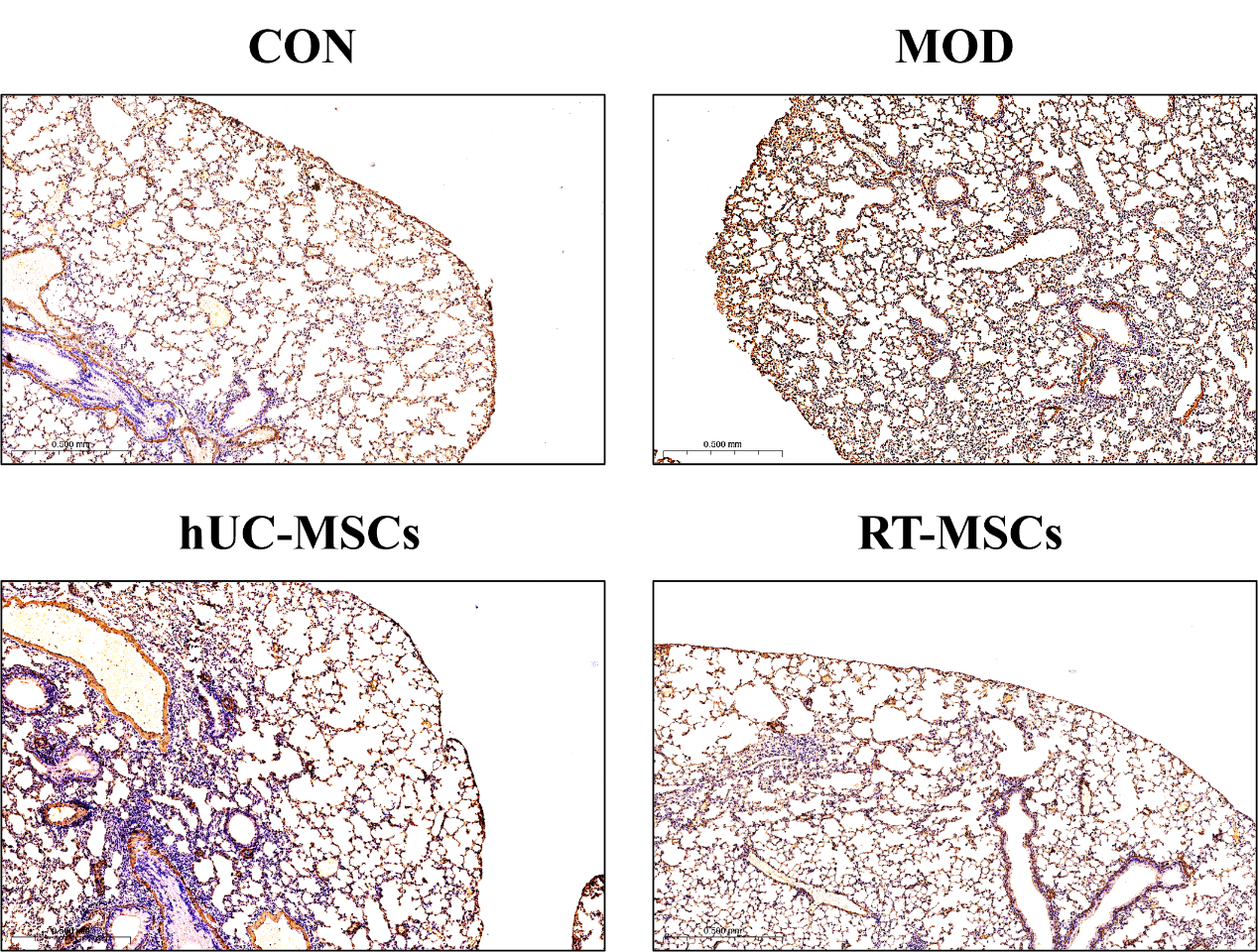
**

Figure. S3. RT-MSC treatment suppresses α-SMA expression in the lung tissues of the fibrosis model.


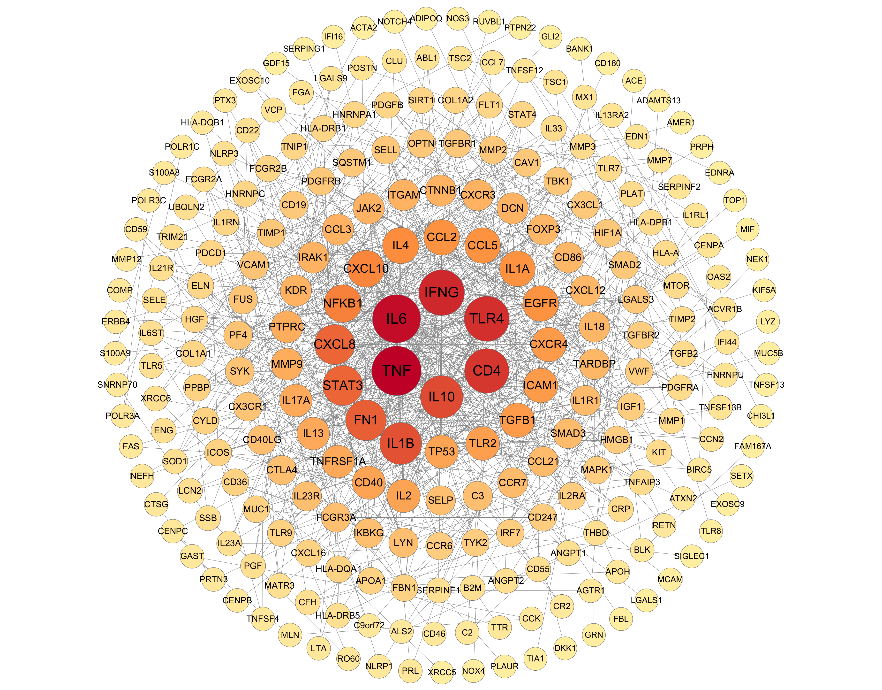


Figure. S4. Disease-related targets identified by searching the OMIM, GeneCards, and DrugBank databases using "Scleroderma" as the keyword were used to construct a protein-protein interaction network, which was then analyzed for its core targets.


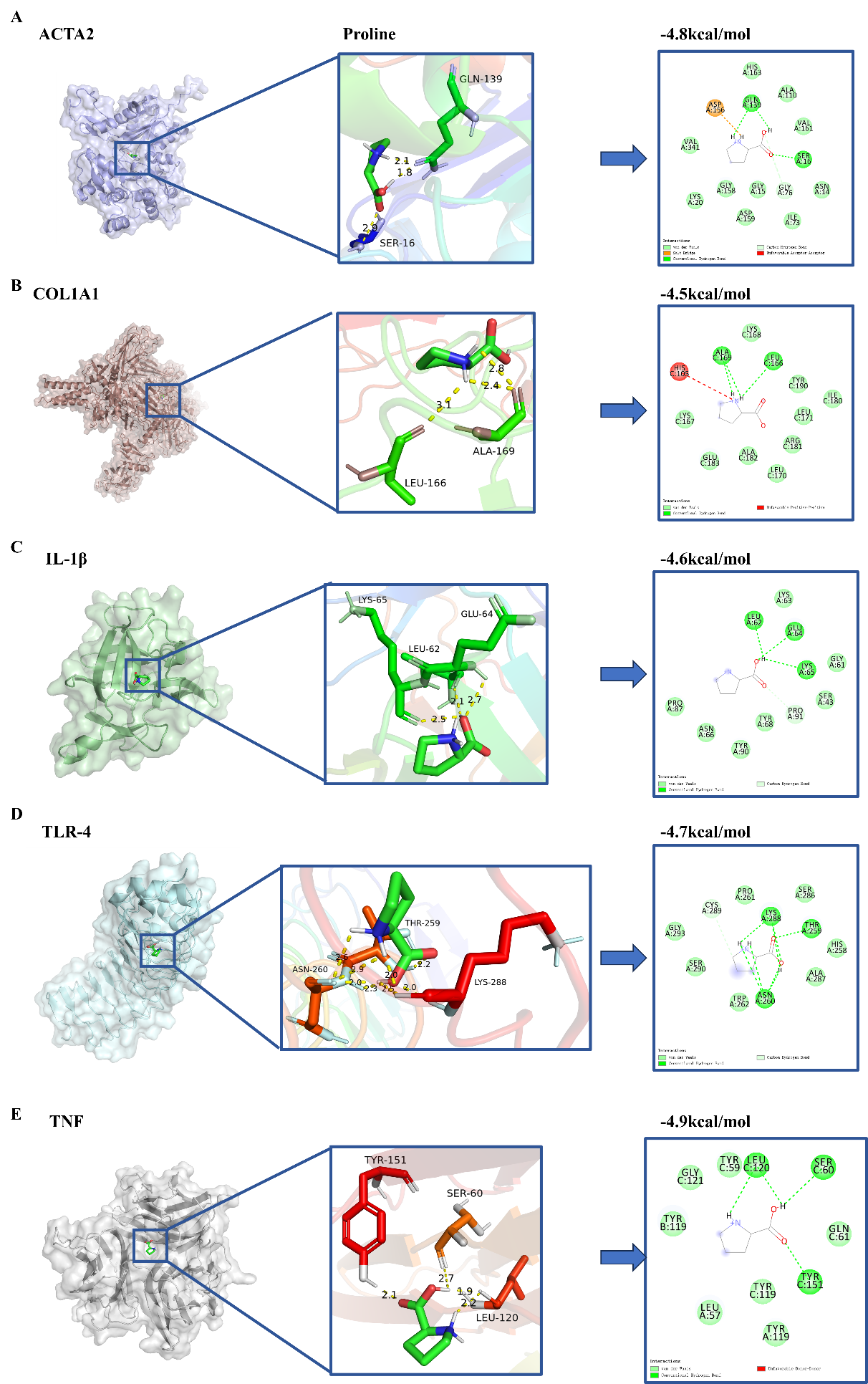


Figure. S5. Molecular docking of proline with ACTA2, COL1A1, IL-1β, TLR4, and TNF. A binding energy less than -4.25 kcal/mol indicates that the compound can bind to the active pocket of the protein.
